# “Like everybody else”: Adolescent perceptions of safe, independent driving with a CHD

**DOI:** 10.1017/S1047951125100784

**Published:** 2025-07-14

**Authors:** Leigh Ann DiFusco, Rachel A. Solomon, Lindsey H. Lee, C. Ward McIntosh, Catherine C. McDonald

**Affiliations:** 1 University of Pennsylvania School of Nursing, Philadelphia, PA, USA; 2 The Children’s Hospital of Philadelphia Center for Injury Research Prevention, Philadelphia, PA, USA; 3 Penn Injury Science Center, Philadelphia, PA, USA

**Keywords:** Adolescent, CHD, driver’s license, learner’s permit, motor vehicle, driving behaviours

## Abstract

**Objective:**

The impact of CHD on safe driving for adolescents is currently unknown. A prospective, qualitative descriptive study was conducted among adolescents with CHD to describe perceived barriers, facilitators, and impacts of CHD on safe, independent driving among adolescents.

**Study design:**

Twenty-eight adolescents aged 15–19 years with CHD participated in virtual, semi-structured interviews in 2023. Adolescent interview data were analysed with conventional content analysis refined by Theoretical Domains Framework in NVivo software.

**Results:**

Mean participant age was 16.4 ± 0.23 years (57% male). Single ventricle physiology (25%) and septal defects (32%) were prevalent diagnoses among the study population. Most participants (92%) did not have driving restrictions.

**Two themes emerged from the data:**

Driving as a normal rite of passage for adolescents with CHD; and confident—but curious—about the impacts of CHD on driving. Adolescents felt confident that driving is not impacted by CHD. They were curious about the likelihood of cardiovascular emergencies and related symptoms while driving. Perceived barriers and facilitators to safe, independent driving were like what has been described in published literature among adolescents without CHD.

**Conclusion:**

These findings celebrate the normalcy of driving during adolescence and reveal curiosities about the impacts of stress, anxiety, fatigue, and risks of heart attack and stroke on driving. Adolescents may look to CHD healthcare providers to help them learn about driving. These findings may inform the development of tools to facilitate meaningful conversations with adolescents regarding driving safety as part of the transition to adult CHD care.

## Introduction

Since 2010, the population of adolescents with CHD has increased by nearly 65%.^1^ Like their peers without CHD, adolescents often seek greater independence from parents or guardians, explore possible life directions and adult roles, and capitalise on newfound freedom.^2–4^ As expected for developmental trajectories, many adolescents with CHD pursue motor vehicle licensure. As an adolescent, obtaining a driver’s license and driving a motor vehicle increases independence through enhanced access to employment and educational opportunities. These effects last into adulthood; however, driving skill relies on visuomotor tasks, cognitive control, and high-level executive functions that are still developing during adolescence.^5–7^ Developmental deficits, lack of driving experience, and unsafe driving practices such as distracted driving may contribute to motor vehicle crashes, a leading cause of death among adolescents in the United States.^6,8–10^ A CHD diagnosis may exacerbate developmental deficits, increasing the risk of motor vehicle crashes during adolescence.^11^

Largely, adolescent transportation studies focus on the broad population and have not considered how a diagnosed CHD impacts participation in graduated driver licensure programs, driving attitudes, and driving behaviours during initial driving experiences. However, early literature examining adults with cardiac disease report impaired driving ability and loss of vehicular control.^12^ Due to advances in medical discoveries and technologies, the population of adolescents with CHD continues to grow. Given the additional risks associated with driving and motor vehicle crashes for adolescent drivers, more evidence is needed to better understand the relationship between driving and CHD.^1^ To address this critical gap, a prospective qualitative descriptive study utilising semi-structured interviews was conducted with dyads including adolescents with CHD and their parents. The purpose was to identify perceived barriers and facilitators to safe, independent driving, and to understand how CHD may impact driving. Results from the adolescent interviews will be reported here.

## Methods

The Children’s Hospital of Philadelphia Institutional Review Board approved this study.

### Sample

The Children’s Hospital of Philadelphia electronic health record was used to generate a list of children aged 15–19 years with a CHD diagnosis. Between December 2022 and October 2023, a member of the study team contacted *n* = 2,252 potential adolescent participants and their parents from the list by email or telephone. In total, *n* = 298 (13%) families expressed interest; of those, *n* = 111 (37%) stopped responding to contact attempts after indicating interest; *n* = 104 (35%) were pre-screened ineligible; *n* = 53 (18%) declined participation; and *n* = 30 (10%) consented to the study. Two were unable to complete the study after enrolling, leaving a final sample of *n* = 28 (9%) adolescents with CHD who completed the study.

Eligible adolescents included those with an ICD-10 code indicative of a CHD lesion (Q20–Q28 range); holding an active learner’s permit, valid driver’s license, or interest in obtaining a learner’s permit or driver’s license; and a parent/guardian willing to take part in the study. All eligible participants had ability to read and write in English; access to a computer, tablet, or smartphone; and provided written electronic informed consent and/or assent (<18 years). Although the results are not reported here, parents of these adolescents were also interviewed (*n* = 29).

### Data collection

Once eligibility was confirmed, participants received a link to complete an electronic demographic and health survey and scheduled the virtual interview. The interview guide was developed based on the refined Theoretical Domains Framework and addressed graduated driver licensure processes, driving experiences, and perspectives of living with a CHD during adolescence.^13^ The interview guide was refined through mock interviews with one adult with CHD and their parent, one CHD healthcare provider, and one nurse researcher specialising in injury research prevention. The interview guide focused on adolescent and parent perspectives of facilitators and barriers towards obtaining a driver’s license and becoming a safe, independent driver. Participants were also asked to consider how CHD could impact the licensure process and driving experiences.

The semi-structured virtual interviews were conducted in 2023 by a PhD-prepared nurse scientist via REDCap and a secure video conferencing system with recording capabilities.^14^ Audio files extracted from the recordings were professionally transcribed, then verified for accuracy and de-identification by a study team member.

### Data analysis

The team developed a codebook based on the refined Theoretical Domains Framework construct domains and emerging concepts in the data. Two authors individually coded 28 adolescent interviews using NVivo 14 with 98.3% intercoder reliability (kappa = 0.86). Codes were organised and grouped to identify patterns and relationships in the data and develop themes. Verbatim examples provided textural description. Demographic data were summarised using descriptive statistics.

### Qualitative data rigour and trustworthiness

Trustworthiness and rigour were addressed based on criteria established by Lincoln and Guba.^15^ Standard operating procedures were established for data collection and analysis. Data were analysed and coded independently by two authors, and then compared to establish dependability and transferability. A third author and current paediatric cardiac healthcare provider reviewed codes to confirm barriers, facilitators, and emerging themes. The qualitative methods, analysis, and results were reported using the PRISMA SRQR to promote transparency and improve rigour, comprehensiveness, and credibility.

## Results

The adolescents in the sample were mostly White non-Hispanic and/or male with a mean age of 16.4 ± 0.23 years old (Table 1). Septal defects (*n* = 9; 32%) and single ventricle physiology (*n* = 7; 25%) were prevalent diagnoses among the study population. More than one type of CHD was reported among 39% (*n* = 11) of participants.

More than half (*n* = 17; 60%) of adolescents had a learner’s permit or driver’s license and most (*n* = 26; 92%) had no driving restrictions. Nine adolescents (32%) reported speaking with CHD healthcare providers about driving. Many adolescents recognised their parents as a primary source of information and support while emphasising the importance of their lifelong connections with CHD healthcare providers.

Two major themes emerged from the data: 1) Driving as a normal rite of passage for adolescents with CHD and 2) confident—but curious—about the impacts of CHD on driving.

### Theme 1: driving as a normal rite of passage for adolescents with CHD

The discussion of driving was largely agnostic to CHD. The safe driving topics mentioned in interviews aligned closely with those found in published literature on adolescents without CHD. Adolescents credited graduated driver licensing programs, driver’s education, positive role models, and adequate practice as facilitators for successful licensure. Distractions from music, mobile devices, and/or other passengers were frequently cited as barriers. Overall, adolescents described safe driving as being responsible, self-confident, and following the rules of the road. Adolescents with CHD seemed motivated to drive safely to keep themselves, others, and their passengers free from harm.

“I feel like their life is – they are in my car, so I don’t want to do anything to harm either of us. We must be as safe as possible, so it’s the same as when it’s just me. I want to be safe when just I’m driving. And, of course, I need to do the exact same things that keep just me safe because I want to keep my friends safe, too (17-year-old female).”

From the adolescents’ perspective, driving was minimally impacted by CHD, even among those with critical CHDs. Most adolescents were excited to drive. Freedom and independence were commonly discussed. In fact, excitement around driving centred on recognising driving as a rite of passage toward increased autonomy.

Most adolescents felt that driving is a normal life experience regardless of having a CHD, and if driving were a concern, their CHD healthcare provider would have addressed it with them. Furthermore, some adolescents seemed surprised by the idea that a CHD might impact driving at all.

“I’ve always been told that I’m like any other kid, other than not being able to play contact sports. They just treat me like it’s normal (16-year-old male).”

“It was very easy [to talk about driving with my CHD healthcare provider] because I do not really have restrictions on driving. It was never really a thing … it was kind of just, oh, congratulations, you got a license, like everybody else… (18-year-old female).”

Few adolescents discussed driving with their CHD healthcare provider. One adolescent described a “quick conversation about the things I’d have to do, and the requirements for it. It wasn’t really a big deal (16-year-old male).” Some parents and CHD healthcare providers were enthusiastic as adolescents pursued licensure. A 17-year-old female shared:

“My parents and healthcare provider are good about driving. They are happy that I can take myself places and be independent, especially graduating this year… [their support has] helped me be more responsible, especially with my new car. It’s been a good experience overall.”

### Theme 2: confident—but curious—about the impact of CHD on driving

Most adolescents spoke confidently about driving and did not think that CHD impacted driving when initially asked. Adolescents seemed curious about driving restrictions or limitations that they may not be aware of; and if, when, and how to address driving with CHD healthcare provider teams.

Concerns about the impact of CHD on their driving were largely derived out of curiosity and what they felt other adolescents with CHD might need to know. When adolescents were asked for advice they might give a peer with CHD about driving, some were unsure due to their own uncertainty regarding the impacts of CHD on driving. Others talked about advising peers with CHD to stay calm behind the wheel despite stressors such as traffic, as stress may increase the chances of a stress-induced cardiac event. One 16-year-old male talked about the importance of being aware of “any possible issues that [a CHD] could cause… If anyone’s in the car, just warn them about that.” Another advised “know where [to] go to pull over in case something happens, or … turn on their four-way blinkers so other cars know that something’s wrong (16-year-old female).”

Adolescents contemplated the possibility of a cardiac emergency while driving due to stress; anxiety symptoms that manifest as tachycardia and palpitations; and fatigue when driving long distances alone. Some even discussed the risk of having a heart attack or stroke because of elevated blood pressure. One adolescent described experiencing mild tachycardia during stressful scenarios, including while driving: “I could just feel my heart rate kind of go up a little bit, sometimes while I’m driving, just elevate a little bit (18-year-old male).”

## Discussion

This interview-based study is the first known to examine perceptions of barriers, facilitators, and impacts of CHD on safe, independent driving among adolescents. Adolescents shared their perspectives about safe driving that focused on normalcy and curiosity. Adolescents in our study were interested in pursuing driving or had successfully obtained a learner’s permit or driver’s license without restrictions. Most felt supported by—but had not discussed driving with—their CHD healthcare providers. Perceived barriers and facilitators were consistent with what has been published in other literature about adolescent drivers.^16–21^

Driving as a rite of passage and celebrating the normalcy of this adolescent stage is also an important dimension of perceived quality of life among adolescents with CHD.^22–24^ Most felt that their experiences were like those of non-CHD peers and that a CHD diagnosis had minimal impact on becoming a safe, independent driver. This sentiment is consistent with what has been reported about health behaviours and everyday living among adolescents with and without CHD.^25–28^

Only one third (*n* = 9; 32%) of adolescent participants—regardless of CHD severity—had discussed driving with a CHD healthcare provider. Adolescents in our study felt that their CHD healthcare provider would have addressed driving with them if it were a concern. CHD healthcare providers play an important role in assessing fitness to drive and counselling adolescents about safe driving behaviours; however, physician visits or health assessments before initial or subsequent noncommercial driver’s licensing is not a consistent requirement among the United States.^29–31^ Adolescents want healthcare providers to help them learn and talk about safe driving.^32^ While conversations about safe driving and licensure may serve as a pathway to transition towards adult care, only 38% of providers surveyed about discussing driving behaviors with adolescents felt prepared to do so, affirming a need for tailored resources that support these conversations,^33,34^ CHD-specific resources could capitalise on self-management skills and emphasise independence gained through licensure to promote key transition components such as understanding and avoiding risky health behaviours; avoiding lapses in medical care; and attending medical appointments independently.^35^

Many adolescents did not think that CHD impacted driving when asked initially, but as the interview progressed, their curiosities evolved. Several adolescents wondered if stress while driving might increase their risk for a heart attack or stroke. While most studies examining crashes and sudden death behind the wheel from cardiac problems focus on adults with acquired cardiovascular diseases, the risk of sudden incapacitation from a heart attack while driving appears to be small.^36,37^ Cardiovascular diseases may have little effect on driving performance and safety.^30^ References to the possibility of heart attack and/or stroke while driving among adolescents in our study highlight a potential knowledge gap and important opportunity for CHD healthcare providers to explore the potential for stress-induced cardiac emergencies while driving with them, even if their risk is minimal.

Adolescents wondered about increased anxiety while driving and if their CHD could impact these symptoms. Driving anxiety among adolescents is variable and may be characterised by a combination of anxious symptomology and avoidance behaviour under certain driving conditions; however, people with CHD—regardless of age or severity—report higher anxiety burdens than non-CHD peers.^38,39^ Driving anxiety may also manifest as stress.^40^ Acute episodes of stress may influence brain regulation and elicit physiologic changes in autonomic reactivity, haemodynamic effects, and vascular function among people with cardiovascular disease.^41^ Practicing mindfulness and relaxation may help alleviate symptoms of anxiety and reduce the chances of stress-induced cardiac emergencies while driving.^42–44^

Fatigue when driving long distances was also concerning for some participants. Sleep disorders or deprivation may increase the risk of motor vehicle crashes while driving.^45^ Adolescent sleep cycles may be shortened or delayed due to pubertal changes and competing social or academic priorities, as well as sleep apnoea and other breathing complications during sleep, which may occur more often with CHDs.^46,47^ Altered sleep may contribute to behavioural, attention and functional difficulties and impact driving behaviours among adolescents with CHD.^48–50^ Interventions that promote sleep health may help alleviate fatigue while driving.^51^

### Limitations

This study was not without limitations. Our sample was comprised of primarily White non-Hispanic adolescents receiving CHD care from a single cardiac centre in a freestanding paediatric acute care hospital in the northeastern United States. These findings should be generalised cautiously to account for differing early driving experiences among adolescents who receive care at other institutions and in other countries. Focusing on specific CHD diagnoses may provide more targeted insight to barriers, facilitators and driving experiences and inform content for driving resources that could enhance driving safety for adolescents with CHD. Additionally, adolescents may not self-report learning difficulties (e.g., attention deficit hyperactivity disorder or the need for an individualised education plan), and these executive function problems may occur secondary to CHD and impact safe driving.

## Conclusion

Adolescents in our study viewed driving like their peers without CHD, as an exciting step towards independence as they transition to adulthood with overall the same perceived barriers and facilitators to safe, independent driving. Parents played a vital role in supporting and normalising their driving experiences. Although many CHD diagnoses pose no immediate dangers while driving, few CHD healthcare providers discussed driving while adolescents expressed curiosity about potential impacts. Adolescents with CHD may benefit from tailored resources and discussions with healthcare providers about physiological symptoms and the potential for cardiac emergencies while driving during routine care to enhance safe, independent driving.

## Figures and Tables

**Table 1.** CHD adolescent demographics (n = 28)

Mean age (years)	16.4 ± 0.23
Gender	(%)
Male	16 (57)
Female	12 (43)
Race/ethnicity^α^	(%)
Black	3 (11)
Other	1 (4)
White	24 (89)
Hispanic or Latino	1 (4)
Not reported	1 (4)
CHD diagnosis^β^	(%)
Aortic anomalies	9 (32)
Arterial anomalies	4 (14)
Atrial Septal Defect	5 (18)
Pulmonary Hypoplasia	1 (4)
Single ventricle	7 (25)
Tetralogy of Fallot	1 (4)
Transposition of the Great Arteries	2 (8)
Valve anomalies	8 (28)
Ventricular Septal Defect	4 (14)
Non-cardiac comorbidities	4 (14)
Spoke with CHD providers about driving	9 (32)
Driving restrictions	(%)
None	26 (92)
Unsure	2 (8)
Licensure status	(%)
Interested in driving (no permit or license)	11 (39)
Learner’s permit	5 (18)
Driver’s license	12 (43)

α Multiple race/ethnicities reported (*n* = 1)

β Multiple CHD diagnoses (*n* = 11).
